# pH Quantification in Human Dermal Interstitial Fluid Using Ultra-Thin SOI Silicon Nanowire ISFETs and a High-Sensitivity Constant-Current Approach

**DOI:** 10.3390/bios13100908

**Published:** 2023-09-27

**Authors:** Yann Sprunger, Luca Capua, Thomas Ernst, Sylvain Barraud, Didier Locca, Adrian Ionescu, Ali Saeidi

**Affiliations:** 1 Xsensio SA, 1015 Lausanne, Switzerland; ali.saeidi@xsensio.com; 2 Nanoelectronic Devices Laboratory, Ecole Polytechnique Fédérale de Lausanne (EPFL), 1015 Lausanne, Switzerland; luca.capua@epfl.ch (L.C.); dlocca@cvcl.ch (D.L.); adrian.ionescu@epfl.ch (A.I.); 3 CEA, LETI, Univ. Grenoble Alpes, F-38000 Grenoble, France; thomas.ernst@cea.fr (T.E.); sylvain.barraud@cea.fr (S.B.); 4 Centre for Cardiovascular Medicine and Devices, William Harvey Research Institute, Queen Mary University of London, London E1 4NS, UK

**Keywords:** pH, biosensor, silicon nanowires, human interstitial fluid, constant current, ISFETs

## Abstract

In this paper, we propose a novel approach to utilize silicon nanowires as high-sensitivity pH sensors. Our approach works based on fixing the current bias of silicon nanowires Ion Sensitive Field Effect Transistors (ISFETs) and monitor the resulting drain voltage as the sensing signal. By fine tuning the injected current levels, we can optimize the sensing conditions according to different sensor requirements. This method proves to be highly suitable for real-time and continuous measurements of biomarkers in human biofluids. To validate our approach, we conducted experiments, with real human sera samples to simulate the composition of human interstitial fluid (ISF), using both the conventional top-gate approach and the optimized constant current method. We successfully demonstrated pH sensing within the physiopathological range of 6.5 to 8, achieving an exceptional level of accuracy in this complex matrix. Specifically, we obtained a maximum error as low as 0.92% (equivalent to 0.07 pH unit) using the constant-current method at the optimal current levels (1.71% for top-gate). Moreover, by utilizing different pools of human sera with varying total protein content, we demonstrated that the protein content among patients does not impact the sensors’ performance in pH sensing. Furthermore, we tested real-human ISF samples collected from volunteers. The obtained accuracy in this scenario was also outstanding, with an error as low as 0.015 pH unit using the constant-current method and 0.178 pH unit in traditional top-gate configuration.

## 1. Introduction

Metabolic acidosis in patients admitted to the intensive care unit (ICU) is commonly associated with various causes and unfavorable outcomes. Studies indicate that the rate of pH change in blood may serve as a better predictor of ICU mortality compared to other metabolic indicators [1,2,3,4]. Local changes in interstitial tissue pH are believed to potentially serve as an early indicator of the onset of shock. Therefore, continuous monitoring of interstitial fluid (ISF) pH could play a critical role in early detection of circulatory shock, reducing ICU mortality and hospital stay, thus providing a relevant impact on health cost. Additionally, the ability to simultaneously monitor pH and other inflammatory markers, such as C-Reactive Protein or lactate, could offer deeper insights into the pathological trajectory of patients and enable more effective therapeutic strategies.

In this context, field-effect transistors (FETs) present excellent potential for the development of wearable and continuous biosensors. They offer advantages such as miniaturization, low power consumption, and the ability to develop label-free bioassays, enabling continuous monitoring. Specifically, fully depleted silicon nanowires (SiNWs) have emerged as promising candidates for integrating a multiplexed biosensing platform [5,6,7,8].

**Figure 1:**
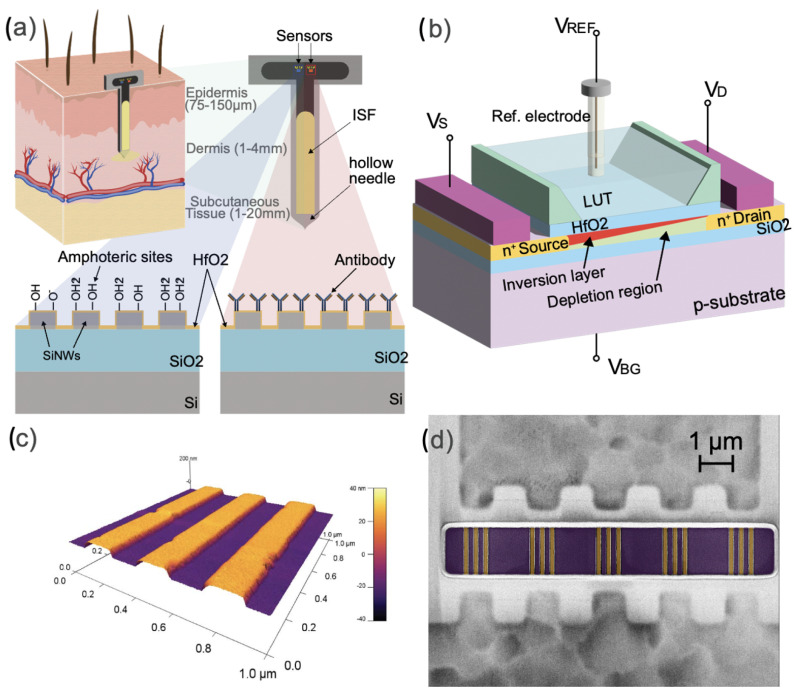
(**a**) Schematic of a continuous and label-free SiNWs pH and protein sensor in human interstitial fluid. (**b**) Measurement configuration of the silicon nanowire Silicon-On-Insulator FET. (**c**) AFM picture of the silicon nanowire FETs used in this work. (**d**) False-colored SEM picture of the silicon nanowire FETs (orange) used in this work.

The objective of our work is to lay the groundwork for the development of wearable and continuous biosensors capable of detecting pH and potentially other biomarkers in human ISF using silicon nanowire transistors (Figure 1a). To achieve this goal, we have devised an optimized constant current method for operating the silicon nanowire transistors. This method offers significant signal amplification and simplifies the readout electronics by enabling measurement of voltage changes in the millivolt (mV) range, instead of the sub-nanoampere (sub-nA) range of current. We compare this new method with the conventional top-gate approach by evaluating the sensitivity of silicon nanowires to pH in ISF-like solutions. Finally, we provide a proof of concept for real human ISF pH sensing by analyzing ISF samples collected from healthy volunteers. The results demonstrate excellent accuracy when compared to a low-volume pH benchtop device (SI 600 from Sensitron).

Previous studies have demonstrated the use of silicon nanowire dual-gate field-effect transistors for ultra-high sensitivity pH sensing [9,10]. However, these studies did not assess the accuracy of the sensors when operating in a complex matrix such as human interstitial fluid. In their work, Dervisevic et al. [11] demonstrated ISF pH sensing on mice, using a polymeric microneedle array-based (PMNA) sensing patch but no testing was performed on human ISF. We believe that our group is the first to demonstrate this new method of operating silicon nanowires for signal amplification, as well as achieving high accuracy in measuring pH in real human ISF samples.

## 2. Materials and Methods

### 2.1. Silicon-On-Insulator Nanowire FETs

The fabrication of silicon nanowire arrays on Silicon-On-Insulator (SOI) substrates have been performed at CEA LETI [12]. In our previous paper [13], we presented the main steps of the fabrication process resulting in arrays of nanowires with a thickness of 30 nm, width of 150 nm and length of 1 µm, connected in parallel. The gate stack is formed of a thin silicon dioxide interface layer, followed by a deposition of 2 nm of high- k dielectric (HfO${}_{2}$). These arrays of robust devices are made in a CMOS-compatible process and are used for the development of our ISF pH sensor.

All electrical measurements were performed on a Keithley 4200A. The silicon nanowires ISFETs were cleaned with 99% ethanol and abundantly rinsed with deionized water before each experiment.

### 2.2. Constant Current Operation

**Figure 2:**
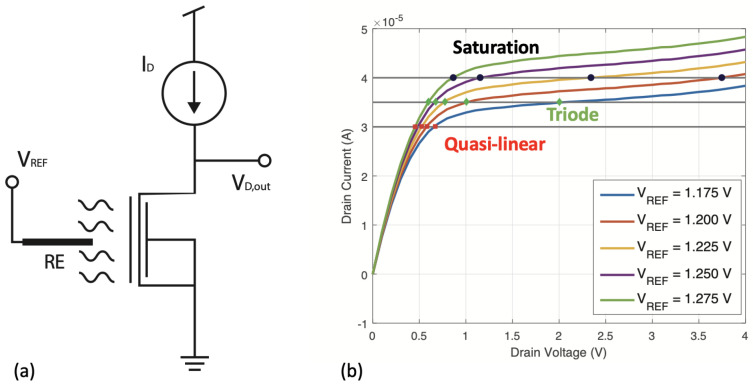
Optimized constant-current method. (**a**) Shows the operating configuration of the transistor. (**b**) Shows four I${}_{D}$-V${}_{D}$ curves at different reference voltage bias. For a given level of constant drain current, the induced changes in the drain voltage when V${}_{REF}$ is changing can be dramatically different, depending on the region of operation. When a constant reference voltage is applied through the Ag/AgCl reference electrode, any change in the pH of the solution results in a variation of the SiNWs conductance. Since the drain current is maintained constant by an external circuit, the transistor compensates the conductance change by adjusting the drain-source voltage.

We propose to investigate a constant drain current operating method for the SOI field-effect transistor, wherein the front gate, back gate, and source terminals are biased with a constant voltage, and the output drain voltage is monitored as the output voltage while the drain current is kept constant (Figure 2a). Our findings demonstrate that the sensor response to pH changes in solution is highly tunable according to the bias conditions of the transistor, thus its operating regime. We compare the linear, triode, and saturated regions (considering the channel length modulation effect) under weak, moderate, and strong inversion conditions determined by the gating voltage, V${}_{REF}$ (Figure 2b). Through our study, we analyzed the behavior of the drain voltage evolution in response to different pH levels across various transistor regimes. The amount of inversion charge in the nanowires’ silicon channels, and consequently the working regime (weak, moderate, or strong inversion), are controlled by the applied reference voltage. Similarly, the operation region of the transistor is determined by the drain-to-source voltage drop and can fall into three categories: linear, triode, or saturation. The behavior of the sensor, including its sensitivity, stability, and power consumption, is significantly affected by both the channel inversion conditions and the operation region.

**Figure 3:**
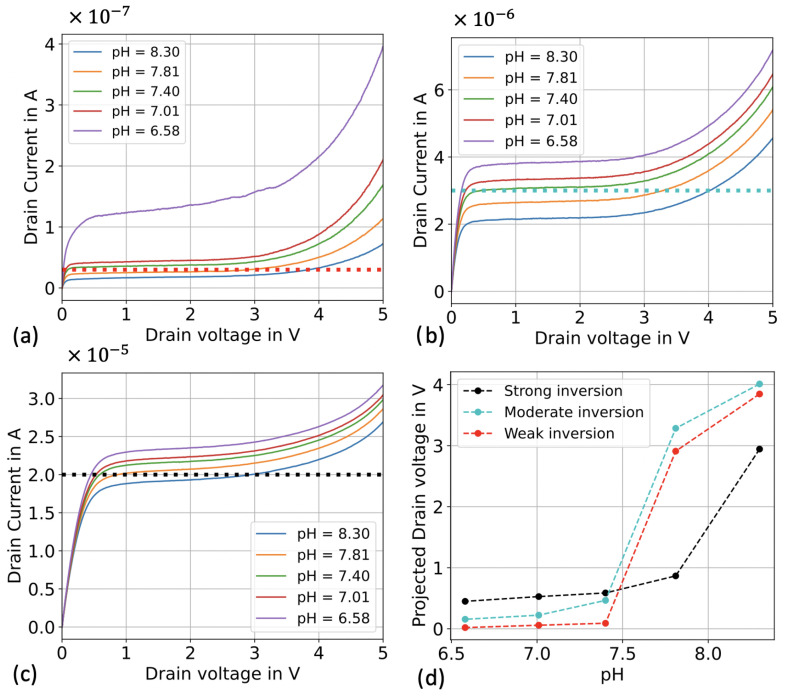
I${}_{D}$-V${}_{D}$ curves at different transistor operating regimes for different pH. (**a**) The transistor is biased in the weak inversion regime. (**b**) The transistor is biased in the moderate inversion regime. (**c**) The transistor is biased in the strong inversion regime. (**d**) Transistor’s sensitivity to pH in the different operating regimes.

Figure 3 presents I${}_{D}$-V${}_{D}$ curves for different working regimes, achieved by varying the applied V${}_{REF}$, and at different pH values. The constant drain current is indicated by horizontal intercepting lines, and the output calibration curve is found to be significantly influenced by the working region. In the case of weak inversion (Figure 3a), the transistor enters the saturation region at very low drain voltage values, resulting in a reduced triode region. This behavior leads to a pH response that predominantly utilizes the saturation region and, to a lesser extent, the breakdown region. Consequently, there is only a small concentration window where the sensor exhibits high sensitivity, reaching up to 6 V/pH. The sensor operating in weak inversion mode demonstrates a high sensitivity as it approaches the saturation region, making it well-suited for use as a threshold sensor in a wide range of applications.

On the contrary, when operating in strong inversion, the SiNWs transistor can function as a pH sensor with a lower sensitivity but an expanded linear range, particularly when biased in the linear operation region (e.g., I${}_{D}$ < 15 μA). Alternatively, by setting the bias drain current to operate the transistor in the saturation region within a chosen pH range (as depicted in Figure 3c), it becomes a highly sensitive non-linear pH sensor. The channel modulation effect has the most significant impact in strong inversion, facilitating the system’s operation in the saturation region. As a result, sensitivity is reduced, but there is an increase in both the extension of the sensing range and overall stability. A straightforward trade-off between stability, the extension of sensing range, and power consumption is offered by the moderate inversion operating regime, shown in Figure 3b.

The creation of an all-region model of the pH sensor while working in constant current operation is not straightforward. When a pH shift occurs in a solution (Δ*pH*), it leads to a charge perturbation on the oxide surface, which, in turn, is counterbalanced by a modification in the channel conductance or carrier density. In our particular use case, where the drain current flowing in the channel is maintained constant, any alteration in conductance triggers an automatic adjustment in the drain voltage. To establish a relationship between the shift in drain voltage and the pH change in the solution, we can express it using the following equation:

(1) $$\frac{\Delta V_{D}}{\Delta pH}=\frac{\Delta V_{G}}{\Delta pH}\frac{\Delta I_{D}}{\Delta V_{G}}\frac{\Delta V_{D}}{\Delta I_{D}}$$

In the ideal scenario, the first factor on the right side of the equation represents the threshold shift resulting from a pH variation, with a maximum of 59 mV/pH which refers to the Nernst limit. The second term is associated with the transistor’s transconductance $g_{m}$, which can be approximated as a constant by biasing the transistor with a fixed reference voltage, since when a pH change occurs, it induces a small signal perturbation on the gate oxide. The last term of the equation is more intricate.

For small signal analysis, this term would reduce to the well-known transistor output resistance, denoted as $r_{0}={\left(\lambda I_{D}\right)}^{-1}$, where λ is the channel-length modulation parameter. However, in this context, the change in drain voltage is too substantial to be treated as a small signal. Yet, in the case of strong inversion and saturation, we can still treat this parameter as a constant, as the I${}_{D}$-V${}_{D}$ relationship exhibits linear behavior (with a constant slope) due to the enforced constant drain current. Hence, the modified equation can be expressed as follows (only if the sensor operates in strong inversion and saturation for the entire tested pH range):

(2) $$\frac{\Delta V_{D}}{\Delta pH}=\beta g_{m}\frac{1}{\lambda I_{D}}$$

The value of β in the equation represents the top-gate pH sensitivity (which is 59 mV/pH in the ideal scenario).

Based on the results obtained, it can be deduced that the weak inversion regime provides the highest sensitivity but within a narrower pH range. On the other hand, the strong inversion regime exhibits the smallest drain voltage shift but operates effectively over a larger pH range. As for the moderate inversion regime, it strikes a favorable balance between the two other regimes by offering nearly linear sensitivity across a practical concentration range. While the weak inversion regime demonstrates superior sensitivity, we have observed a decrease in sensor stability when operating within it. Therefore, for the experiments conducted in the next section, we will primarily utilize a sensor operating in the moderate inversion regime. This choice is driven by its ability to provide an optimal trade-off between sensitivity, sensing range, and stability.

### 2.3. Sample Preparation and ISF Collection

**Figure 4:**
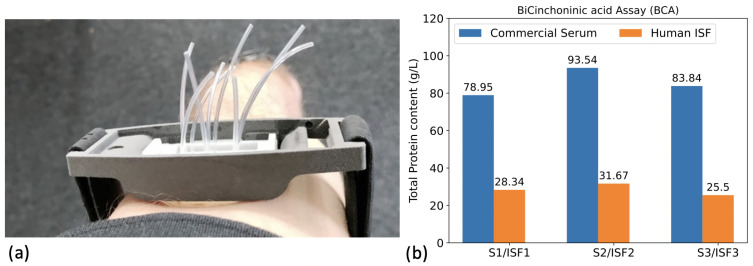
(**a**) Picture of the device used to collect human ISF. The collector consists of a rigid support with micro-needles directly connected to Tygon tubes. (**b**) Total protein content assessed by BCA assay on (i) the commercial sera samples (**S1**), (**S2**) and (**S3**) and (ii) on ISF of the three human healthy volunteers.

To evaluate the pH sensitivity of the silicon nanowire sensors, we prepared five solutions with pH values of 6.58, 6.85, 7.08, 7.47, and 7.88 in HEPES and MES buffers. These solutions are referred to as the “buffer” set. Additionally, we prepared five solutions with pH values of 6.52, 6.87, 7.16, 7.52, and 7.96 in diluted human sera, which are referred to as the “ISF-like” set. For the buffer set, we used HEPES buffer at a concentration of 150 mM for the pH range between 6.85 and 7.88, and MES buffer at the same concentration for the 6.58 sample. To adjust the pH, we spiked the samples with NaOH 150 mM or HCl 150 mM while maintaining constant ionic strength. The ISF-like samples were adjusted using a similar method. These samples were prepared by diluting a pool of human sera (Hytest CRP-free serum) three times in PBS 1X at 150 mM. The dilution factor of three was chosen to replicate the concentration of total protein content typically found in human interstitial fluid, which accounts for approximately one-third of the total plasma protein content [14,15,16]. In our study, we confirmed the 3-fold dilution factor of the total protein content in real human ISF with respect human serum by using the BiCinchoninic acid assay (BCA) on three distinct collected ISF samples (Figure 4b).

The selected pH range corresponds to what is believed to be the relevant range for interstitial fluid, as suggested by clinicians and literature, which considers the interstitial fluid pH to vary between approximately 6.5 and 8 under physiopathological conditions [17,18]. Additionally, three other ISF-like solutions were prepared using three different pools of sera with varying total protein content (Figure 4b) (BBI CRP-depleted human serum (**S1**), Human serum H4522 (**S2**), and 6914 from Sigma-Aldrich (**S3**). These samples were diluted three times in PBS 1X at 150 mM. We tested these samples on the same silicon nanowire sensors, and their pH values were determined by back-calculation using the calibration curve obtained from the first set of ISF-like samples. The pH values obtained were then compared to those obtained using a benchtop pH meter (Fisherbrand accumet AE150).

Finally, real human ISF samples were collected from three different healthy volunteers (Figure 4a). Each volunteer provided approximately 20 µL of ISF, which were pooled together to obtain a total volume of approximately 60 µL. The pH of the pooled ISF samples was measured using a low-volume benchtop pH meter (SI 600 from Sensitron). A calibration curve using four solutions of ISF-like samples was generated on a new sensor, and the pH of the pooled ISF samples was assessed in both top-gate and constant-current configurations.

## 3. Results

### 3.1. pH Sensing in Top-Gate Configuration

**Figure 5:**
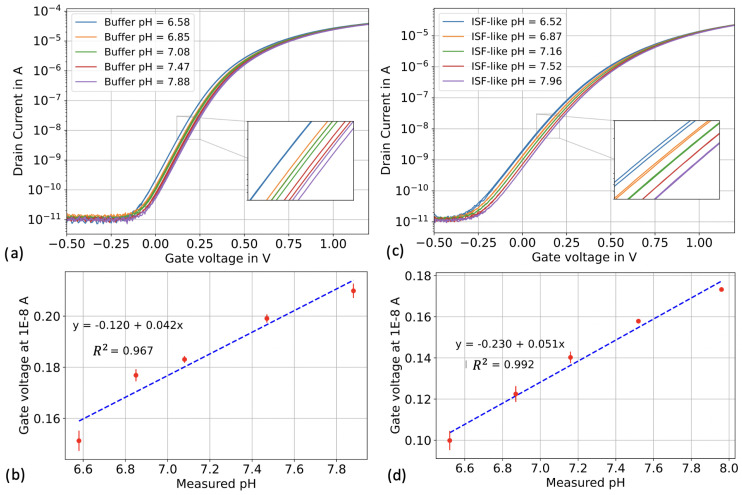
pH calibration in top-gate configuration for buffer and ISF-like sets. Each calibrator was repeated three times. (**a**) I${}_{D}$-V${}_{R}EF$ in buffer calibrators. (**b**) I${}_{D}$-V${}_{R}EF$ in ISF-like solutions calibrators. (**c**) Linear fitting in buffer calibrators. (**d**) Linear fitting in ISF-like solutions calibrators.

To compare the pH sensitivities of the silicon nanowires in both “buffer” and “ISF-like” matrices, we conducted voltage sweeping experiments in top-gate configuration. The reference voltage was swept from −0.5 V to 1.5 V using an external Ag/AgCl reference electrode immersed in the liquid under test. The applied back-gate voltage (V${}_{BG}$) was 0 V, the source voltage (V${}_{S}$) was 0 V, and the drain voltage (V${}_{D}$) was 0.5 V. We first exposed the sensors to the five different pH solutions from the buffer set, followed by the five different pH solutions from the ISF-like set. Figure 5a,c illustrates the transfer characteristics of both the buffer and ISF-like solutions in a semi-logarithmic scale.

We observed that the subthreshold slope was degraded when the sensors were exposed to the ISF-like solutions. We believe that this degradation is a result of the high concentrations of proteins and metabolites present in the ISF-like solutions, which can adsorb onto the sensors. These adsorbed non-specific analytes may carry charges or become polarized, leading to the formation of an additional capacitor on top of the gate dielectric. This can deteriorate the gate coupling capability of the transistor. Let’s mention that the subthreshold slope can be “recovered” after extensive washing with ethanol 99% and deionized water, as shown in the supplementary material (Figure S1). Despite the degradation in subthreshold slope, the transistor’s drain current ON/OFF ratio, exceeding 5 orders of magnitude, remained consistent in both buffer and ISF-like solutions. Additionally, we observed minimal sensor hysteresis in both sets of solutions.

To determine the pH sensitivity, a reference voltage at 10 nA was extracted for each pH value. The sensors exhibited a sensitivity of 42 mV/pH in buffer and 51 mV/pH in ISF-like matrix (Figure 5b,d). Extensive testing of different silicon nanowires from this batch of chips revealed an average sub-Nernstian pH sensitivity of 40 mV/pH (±7 mV) in buffer. The reason for the observed increase in sensitivity in the ISF-like matrix is unclear and requires further investigations. Nevertheless, the sensors demonstrated high reliability in pH measurement in both matrices, with minimal variation between the three repeats of each calibration sample. The calibration curves in Figure 5b,d exhibited a good linear relationship between pH value and reference voltage both in buffer ($R^{2}=0.96$) and in ISF-like solutions ($R^{2}=0.99$).

Following the sensitivity assessment in voltage sweeping mode, we proceeded to real-time pH sensing in top-gate configuration. The reference voltage was biased at 0.2 V, the drain voltage at 0.5 V, and both the back gate and the source were biased at 0 V. The drain current was recorded over time. The ISF-like calibrators were injected for 120 seconds, forming a “pyramid” as the pH transitioned from high to low. One of the calibrator samples was retested after the pyramids to assess the accuracy of the calibration curve. Subsequently, three different ISF-like samples with unknown pH (**S1**, **S2** and **S3**, derived from distinct pools of sera (BBI, H4522, and H6914), were injected for 120 seconds. The pH of these “unknown” samples was back-calculated using the pH calibration curve and compared to the readings from the benchtop pH meter.

**Figure 6:**
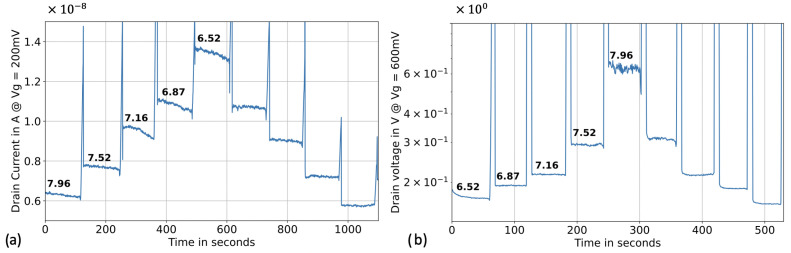
Real time pH sensing in human interstitial fluid-like solution. (**a**) pH sensing in top-gate configuration, where the reference voltage was biased at 0.2 V, the drain voltage at 0.5 V and both the back gate and the source were biased at 0 V. (**b**) pH sensing in optimized constant-current method with a reference bias of 0.6 V, a forced drain current of 2 µA and a source and back gate bias of 0 V.

The silicon nanowire sensor exhibited a clear pyramidal behavior with a small level of hyteresis (Figure 6a). When back-calculating the unknown samples using the calibration curve, we achieved excellent accuracy. The error between the values obtained from the benchtop pH meter and the silicon nanowire sensors was below 2% (Table 1). The demands for higher precision, robustness, and real-time sensing of pH and proteins in critically ill patients in the ICU are challenging to meet with classical top-gate methods and motivated the constant-current method used in the next section.

### 3.2. pH Sensing in Constant Current Operation

The same silicon nanowire sensors were measured in the optimized constant-current method, following their evaluation in top-gate configuration. The experimental protocol followed a similar procedure: we generated a pyramid in the ISF-like solution, established a calibration curve, and tested the unknown samples. The transistors were operated within the moderate inversion regime, employing a reference bias of 0.6 V, a fixed drain current of 2 µA, and a source and back gate bias of 0 V. The drain voltage was recorded over time.

In this configuration, the silicon nanowire sensor exhibited a non-linear pH sensitivity, with a sensitivity exceeding 400 mV/pH and minimal hysteresis (Figure 6b). The back-calculated values of the unknown pH samples demonstrated improved accuracy compared to the top-gate configuration, with a maximum error below 1% (Table 1). The relationship between pH and the drain voltage appeared to follow an exponential trend rather than a linear one. Consequently, an exponential fitting method was employed for the calibration curve.

### 3.3. pH Sensing in Real Human ISF

#### 3.3.1. Protocol

Following ethical committee approval, human interstitial fluid (ISF) was collected from three healthy volunteers, similarly to other works [19,20]. The collector was placed in between the biceps and triceps of the volunteers (Figure 4a). Approximately 20 µL of dermal ISF was sampled on each volunteer and protein total concentration was assessed using a BCA (Figure 4b). The samples were then pulled together in order to obtain one test sample of 60 µL, referred to as “unknown”. We took the decision of pulling the ISF samples based on the minimal volume, required by the benchtop pH meter (SI 600 from Sensitron), to have an accurate measurement.

#### 3.3.2. Measurement

To assess the pH accuracy of our SiNWs platform in real human ISF, we prepared four ISF-like calibration solutions following the method described in the previous section. For each testing method, we generated a calibration curve using these solutions and subsequently tested the “unknown” ISF sample. All experiments were conducted using the same SiNWs sensor.

**Figure 7:**
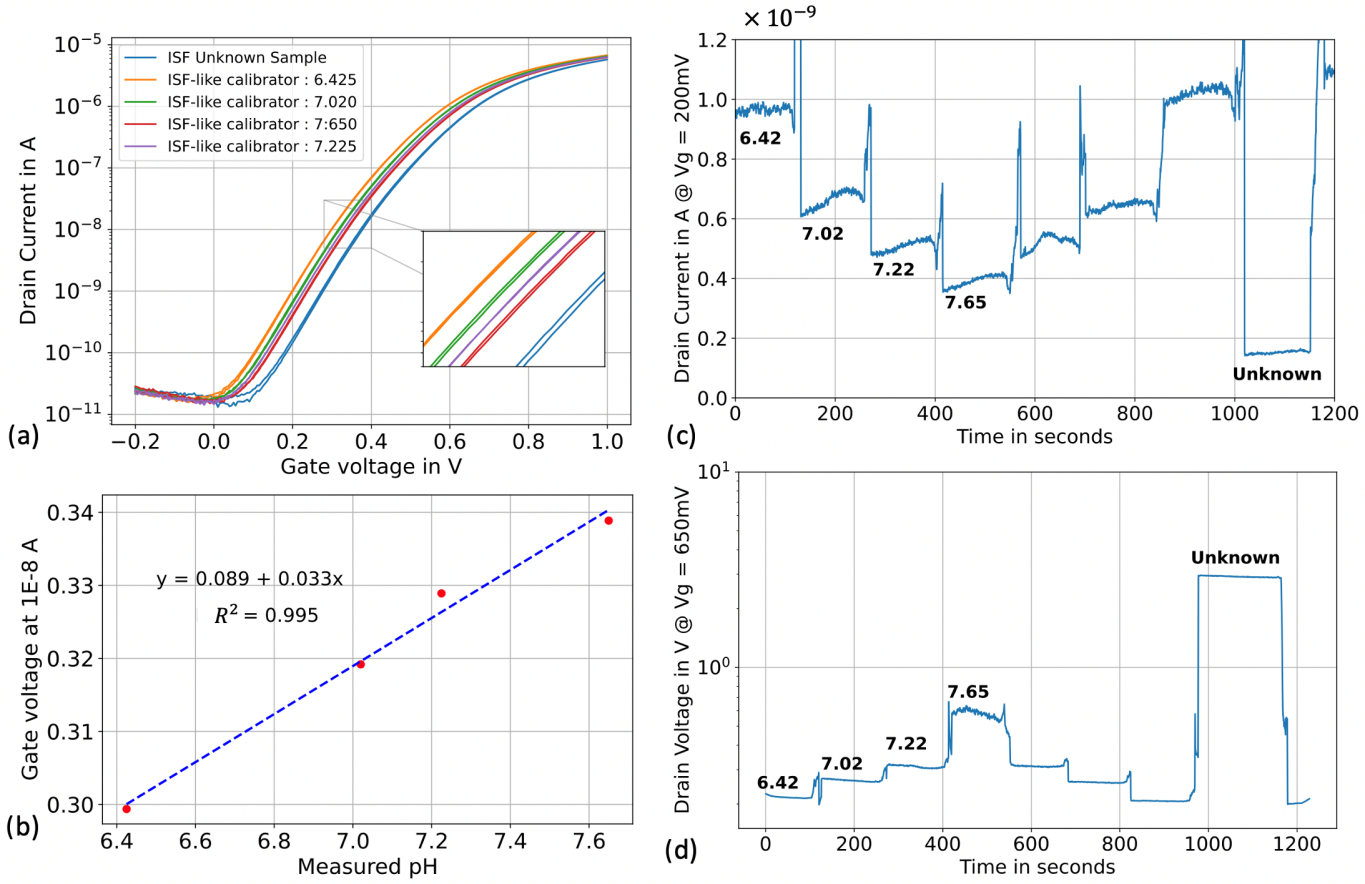
pH sensing in real human ISF (**a**) ID-VREF in ISF-like solutions calibrators and with the real human ISF sample. (**b**) Linear fitting in ISF-like solutions calibrators. (**c**) pH sensing in top-gate configuration, where the reference voltage was biased at 0.2 V, the drain voltage at 0.5 V and both the back gate and the source were biased at 0 V. (**d**) pH sensing in optimized constant-current method with a reference bias of 0.65 V, a forced drain current of 5 µA and a source and back gate bias of 0 V.

Initially, we tested the SiNWs in voltage sweeping mode in top-gate configuration. In ISF-like solutions, we observed a lower sensitivity (Figure 7a) compared to the previous set of experiments (33 mV/pH vs. 51 mV/pH). However, the sensors exhibited a linear response ($R^{2}$ > 0.99) and we backcalculated the value of the “unknown” ISF sample using linear fitting (Figure 7b).

Following the voltage sweeping test, we proceeded to real-time pH sensing in top gate configuration. We followed the same protocol as described in the previous section, injecting the ISF-like calibrators for 120 seconds to form a pyramid shape, followed by injection of the “unknown” ISF sample (Figure 7c). The pH value of the “unknown” sample was back-calculated using a “log-lin” fitting. Finally, we used the constant-current operation method following the same protocol. The transistors were operated within the moderate inversion regime (Figure 7d).

The results of this section are summarized in Table 2. Back-calculating the pH of the ISF sample using both top-gate methods yielded values of 8.79 for the voltage sweeping method and 8.78 for the real-time method. The pH value of the ISF sample measured by the benchtop pH meter was 8.60. As calculated in the previous section, the error percentage was slightly above 2%. Once again, with an outputted pH value of 8.61, the constant-current operation method demonstrated significantly better accuracy, with an error percentage as low as 0.2%.

Surprisingly, the pH of the pooled human interstitial fluid sample was notably higher than anticipated, measuring at 8.60. Previous works have measured pH in skeletal muscles interstitial fluid using microdyalisis and found values between 6.9–7.4 [21], others claim that ISF pH should vary between 6.6–7.60 and can rise up to 8 [17,18].

Table 3 summarizes our experimental results versus existing reports in the the state of the art literature [9,11,22,23]. Our methods provides sensitivies comparable or better than the existing method, with the unique advantage of having some of the ever reported accuracy in the full ISF matrix, which to our best knowledge was not reported in other works. Overall, it appears that silicon nanowires SOI FETs, operated in constant current configuration, form a sensing platform meeting miniaturization and performance criteria for pH integrated sensing of human interstitial fluid.

## 4. Conclusions

In summary, our study presents a novel approach to use silicon nanowires ISFETs as high-sensitivity pH sensors for wearable applications. By fixing the current bias of silicon nanowires and monitor the resulting drain voltage as sensing signal, we were able to outperform the accuracy of conventional top-gate methods. We provided a phenomenological explanation on how this constant current method works and what parameters influence the sensing performance. It was demonstrated that by fine tuning the injected current levels and operating the transistors in different regimes, we can optimize the sensing conditions according to different sensor requirements. Typically, we deduced that the weak inversion regime demonstrates superior sensitivity but poor stability and sensing range, while the strong inversion regime shows poorer sensitivity but superior stability and sensing range. We established that the moderate inversion regime provided the optimized balance for pH sensing by offering high sensitivity and good stability across a practical pH range. Additionally, our proposed approach simplififes the requirements of the wearable readout circuit, by enabling voltage measurement instead of current at nanoamper range.

In our study, we found that human serum diluted three times in PBS 1X exhibited a similar total protein content to human ISF. We validated this threefold difference in total protein content by conducting BCA assays on three different human ISF samples. Using the three times diluted human serum as ISF-like calibrators, we successfully measured pH in ISF-like solutions with high accuracy (0.07 pH unit) through a constant current method, which outperformed conventional top-gate methods (0.13 pH unit). Subsequently, we applied the constant current method to real human ISF sensing, obtaining a pH value of 8.60 for the pooled human ISF sample. This value was notably higher than what is typically reported in the literature for ISF in general, making us the first group to measure dermal interstitial fluid pH using silicon nanowire ISFETs. Further research is needed to explore the pH baseline and dynamics of dermal human interstitial fluid, as well as to assess the long-term stability and potential applications of our constant current method for continuous and real-time biosensing.

## Figures and Tables

**Table 1:** Comparison between benchtop pH meter, top-gate configuration method, and optimized constant-current method for the “unknown” samples. The error is measured, in %, as the difference between the pH meter reading and the other methods reading normalized by the pH meter reading.

Sample	pH Meter Reading	pH SiNWs in Top-Gate	Error %	pH SiNWs in Constant Current Operation	Error %
ISF-like S1	7.61	7.68	0.92	7.68	0.92
ISF-like S2	7.59	7.68	1.19	7.62	0.40
ISF-like S3	7.62	7.75	1.71	7.66	0.52

**Table 2:** Comparison between benchtop pH meter, top-gate configuration methods, and optimized constant-current method for the “unknown” human ISF sample.

Method	pH Meter Reading	pH Measured	Error %
Top-gate sweeping	8.60	8.79	2.2
Top-gate real-time	8.60	8.78	2.2
Constant-current	8.60	8.61	0.2

**Table 3:** Comparison of pH sensing sensitivity and accuracy in different matrices.

Sensor	Method	Substrate	Sensitivity (mV/pH)	Accuracy (pH Units)	Matrix
[11]	Potentiometry	Polyaniline-coated PMNA	62.9	±0.036	Artificial ISF buffer
[9]	Dual-Gate ISFETs	Silicon nanowires	938.4	N.A	pH calibrators
[22]	Potentiometry	Hydrogen selective membrane (HSM)	>50	±0.3	Euthanized rat ISF
[23]	Constant current	Silicon nitride	41	N.A	pH calibrators
This work	Top-gate ISFETs	Silicon nanowires	42 ± 10	±0.18	Human ISF
This work	Constant current	Silicon nanowires	≈400	±0.01	Human ISF

## Data Availability

Not applicable.

## Supplementary Materials

The following supporting information can be downloaded at: https://www.mdpi.com/article/10.3390/bios13100908/s1, Figure S1: pH calibration in top-gate configuration before and after contact with ISF-like solutions. (a,b) ID-VG calibration curves before and after contact with ISF-like solutions for two sensors. The sensors are cleaned with 99% ethanol and abundantly rinsed with DW both before after being challenged with ISF-like solutions and after. (c,d) pH sensitivity extracted at 1nA, we observe no impairement after the use of ISF-like solutions; Figure S2: Four replicates of pyramids in ISF-like solutions both in top-gate and constant current. (a,b) Top-gate configuration, the Vref was biased at 100 mV. Lowest value in current is pH 8.08. The higher the signal, the higher the CV%. (c,d) Constant-current configuration, the transistors are biased in the moderate inversion regime. Highest value in drain voltage is pH 8.08.

## Abbreviations

The following abbreviations are used in this manuscript:

BCA	BiCinchoninic acid Assay
ICU	Intensive Care Unit
ISF	Interstitial Fluid
ISFETs	Ion-Sensitive Field-Effect Transistors
PBS	Phosphate-Buffered Saline
pH	Potentiel of hydrogen
SiNWs	Silicon Nanowires
