# Supplementary material for: pH Quantification in Human Dermal Interstitial Fluid Using Ultra-Thin SOI Silicon Nanowire ISFETs and a High-Sensitivity Constant-Current Approach
Source: Biosensors (Basel). 2023 Sep 27;13(10):908. doi: 10.3390/bios13100908 (PMC10605508; doi:10.3390/bios13100908)
Supplement: Supplementary file 1 [file biosensors-13-00908-s001.zip › biosensors-2569731-supplementary.pdf]

pH Quantification in Human Dermal Interstitial Fluid Using Ultra-Thin SOI Silicon Nanowire ISFETs and a High-Sensitivity Constant-Current Approach

Y. Sprunger[1,2]* and L. Capua[1], T. Ernst[3], S. Barraud[3], A.M. Ionescu[1] and A. Saeidi[2]
[1]Nanoelectronic Devices Laboratory, EPFL, 1015, Lausanne, Switzerland
[2]Xsensio SA, 1015, Lausanne, Switzerland.
[3]CEA, Leti, Univ. Grenoble Alpes, F-38000 Grenoble, France

Supplementary information:

The pH sensitivity of two clean SiNWs was assessed, in top-gate sweeping mode, using three pH **commercial calibrators** (pH 5.0, 6.86 and 9.18). The sensors were then challenged with ISF-like solutions (see figure 2.), cleaned with 99% ethanol, abundantly rinsed with DW and retested for pH sensitivity. The pH sensitivity as well as the subthreshold slope were not impaired by the contact with three-times diluted human serum.

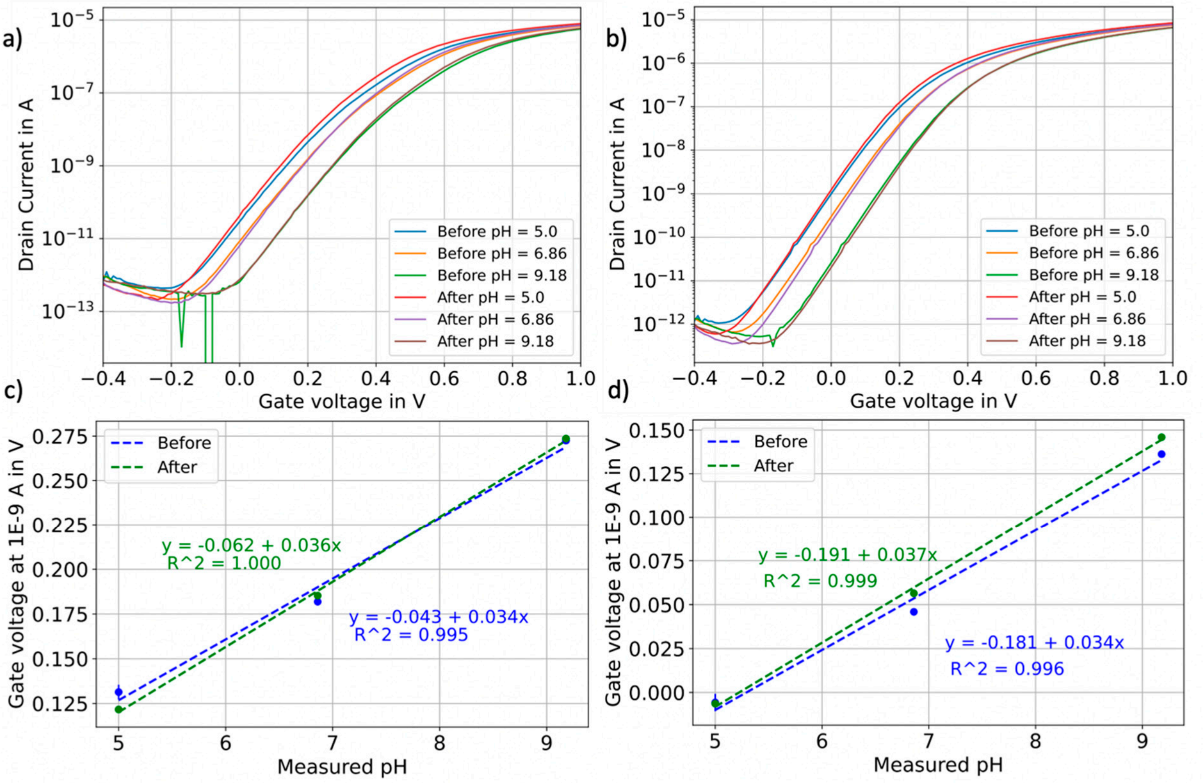

Figure S1. pH calibration in top-gate configuration before and after contact with ISF-like solutions. a,b) ID-VG calibration curves before and after contact with ISF-like solutions for two sensors. The sensors are cleaned with 99% ethanol and abundantly rinsed with DW both before after being challenged with ISF-like solutions and after. c,d) pH sensitivity extracted at 1nA, we observe no impairement after the use of ISF-like solutions.

The sensors were then challenged with three **ISF-like solutions** at different pH in order to generate four pyramids. Both in top-gate configuration (fixed Vref, looking at ID as output) and with our constant current method (in the moderate inversion regime). We have 8 replicates for pH 7.59 and four replicates for pH 8.08 and 7.02. Boxplots with coefficient of variation (std/mean) are shown for both top-gate and constant current method.

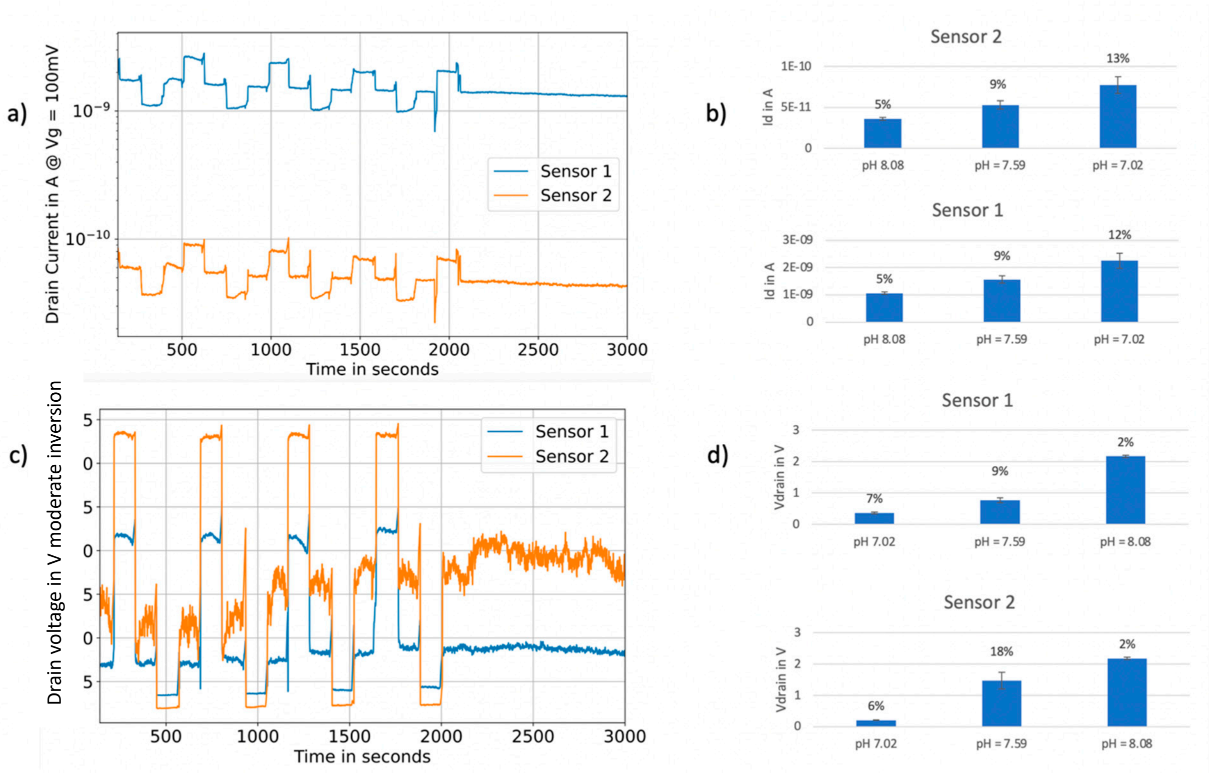

Figure S2. Four replicates of pyramids in ISF-like solutions both in top-gate and constant current. a,b) Top-gate configuration, the Vref was biased at 100mV. Lowest value in current is pH 8.08. The higher the signal, the higher the CV%. c,d) Constant-current configuration, the transistors are biased in the moderate inversion regime. Highest value in drain voltage is pH 8.08
